# IRF2BPL gene variants with dystonia: one new Chinese case report

**DOI:** 10.1186/s12883-023-03077-x

**Published:** 2023-01-21

**Authors:** Fei Yang, Hui Li, Yi Dai, Ran Zhang, Jiang-tao Zhang

**Affiliations:** 1 Department of Neurology, Chengde Central Hospital, No. 11 Guangren Street, Shuangqiao 067000 Chengde, China; 2 Chengde Medical University, Chengde, China; 3 Department of Neurology, Peking Union Medical College Hospital, Chinese Academy of Sciences & Peking Union Medical College, Beijing, China

**Keywords:** Dystonia, Dysarthria, IRF2BPL mutation

## Abstract

**Background:**

The carriers of damaging heterozygous variants in interferon regulatory factor 2 binding protein-like (IRF2BPL), encoding a member of the IRF2BP family of transcriptional regulators, may be affected by a variety of neurological symptoms, such as neurodevelopmental regression, language and motor developmental delay, seizures, progressive ataxia and a lack of coordination, and even dystonia.

**Case presentation:**

We report a Chinese boy who presented with dystonia, dysarthria, and normal development due to nonsense IRF2BPL mutation, with intact imaging and EEG findings but without developmental delays or seizures. Whole-exome sequencing revealed a novel nonsense variant IRF2BPL (NM_024496) Exon C.562C > T (p.Arg188*).

**Conclusion:**

This case report presents a Chinese boy with a novel nonsense variant in IRF2BPL, displaying rapid progressive dystonia and dysarthria, without early developmental delay or epilepsy; expands the IRF2BPL phenotypes in the Chinese population; and raises awareness of patients with IRF2BPL.

**Supplementary Information:**

The online version contains supplementary material available at 10.1186/s12883-023-03077-x.

## Background

The complete loss and overexpression of interferon regulatory factor 2 binding protein-like (IRF2BPL) are both lethal, while a partial knockdown leads to neurodegeneration [1]. IRF2BPL mutation is mainly manifested as seizures in the Chinese population [2], and we report a boy who presented with dystonia.

## Case presentation

This 10-year-old boy, who had non-consanguineous healthy parents, presented with an occult onset with progressive aggravation mainly in dystonia and dysarthria. His family members did not have a similar disease, and he was born from an uneventful pregnancy with normal delivery.

The boy had met all developmental milestones until age 8 years, when he experienced motor regression with abnormal posture when walking, followed by frequent falls, dysarthria, and drooling. The patient’s clinical symptoms developed progressively, especially in his motor and language abilities. One year later, he had lost walking skills; however, he could maintain a sitting position for a short time, without epileptic seizures. He was also sitting on the sofa at the latest follow-up visit; however, his muscle strength had not changed since the previous visit, and he could stand by himself for tens of minutes. His language ability had deteriorated significantly, with severe dysarthria, but he had normal comprehension and could communicate with family members. At the same time, the patient had difficulty urinating, manifested as prolonged urination time. His mental development was normal, with a Mini-Mental State Examination score of 26/30.

His general physical examination was unremarkable, and his neurological examination revealed a cooperative and alert boy who was oriented to person and place, while his speech was slow and dysarthric but intelligible. The remainder of the cranial nerve examination was normal. The motor exam of the bedridden boy showed symmetric decreased strength (4/5) and tone in his extremities, mild disuse atrophy in muscle bulk, abnormal posture of both hands, and contracture of both wrist and ankle joints, while deep reflexes were marked, with ankle clonus. The cardiorespiratory and abdominal examination was normal, and the results of the neuropsychological assessment at age 8 years were normal.

The magnetic resonance imaging (MRI) features of the brain were normal and did not present atrophy (Fig. 1, see Additional file 1). The electroencephalogram (EEG) was normal and did not show spike wave characteristics (Fig. 2, see Additional file 1).

Whole-exome sequencing (QIAamp DNA Blood Mini Kit) was performed on the NextSeq 500 or HiSeq 2500 sequencing platform. It revealed a novel nonsense variant IRF2BPL (NM_024496) Exon C.562C > T (p.Arg188*). The mutation was not reported in the population database. It caused the protein to have a stop code at position 188, resulting in termination. However, whole-exome sequencing did not reveal any mutation in the parents, and we accepted it as a new mutation.

## Discussion and conclusions

We described the first case of a Chinese patient presenting with dystonia, dysarthria, and normal development due to nonsense IRF2BPL mutation, with intact imaging and EEG findings but without early developmental delays or seizures. The clinical features, as previous IRF2BPL mutation cases reported, include various epilepsy syndromes, developmental delay, cerebral atrophy shown by neuroimaging, and abnormal EEG [3–5]. Antonelli F et al. reported a case of familial dystonia which characterized by dystonic and ataxic progressive syndrome due to a mutation in the IRF2BPL [3], but the proband’s father, two paternal uncles, and paternal grandmother did not receive whole-exome sequencing because they had died. Meanwhile, 10 other patients similar to ours [4, 5] had the clinical manifestations of dystonia, ataxia, hyperreflexia to various degrees, and epilepsy (Table, see Additional file 2).

The main etiology for dystonia includes trauma, infections, exposure to medications, and gene mutations (dystonia-associated genes, including KMT2B, VPS16, HPCA, KCTD17, DNAJC12, SQSTM1, IRF2BPL, YY1, and VPS41) [4]. Marcogliese conducted fruit fly experiments and confirmed that IRF2BPL mutation led to a range of neurological phenotypes [3]. A previous case study described a 36-year-old woman (patient 1) whose brain iron concentration diffused mildly in T2-weighted imaging, which might cause dystonia and ataxia [5]; similar findings described neurodegeneration with brain iron accumulation. Therefore, it is intriguing to speculate that increasing iron deposits would relate to IRF2BPL dysfunction. Of note, there was a man (patient 8) who presented with dystonia that was associated with nigrostriatal degeneration; the patient presented with a symmetric, uniform reduction of striatal dopamine transporter density [6]. However, further studies are needed to elucidate the role of dystonia in IRF2BPL mutation carriers.

Among the nine individuals, seven patients (1,2,3,4,6,7 and 10) presented with epilepsy, and six patients (1, 2, 5, 6, 7, and 8) showed brain atrophy. A recent case study of a 24-year-old patient showed epilepsy in adulthood (patient 10) [7]. Our patient may be in the latent period of epilepsy, and he may present brain atrophy in the next few years, but follow-up is needed to verify whether these symptoms will appear in our patient. Moreover, other patients (1, 5, and 7) progressed relatively mildly in contrast to our patient, with an average time of several years.

The distal gene mutations that close the C-terminal RING finger of the IRF2BPL protein may cause a more severe phenotype [8]. However, our patient presented with dystonia and dysarthria, and the mutation site may be further away from the distal end. In addition, it may be related to the expression level of the IRF2BPL gene.

In conclusion, we present a new case with IRF2BPL mutation manifesting with dystonia, ataxia, dysarthria, and normal development. This expands the heterogeneous IRF2BPL phenotypic spectrum in the Chinese population.

## Patient perspective

As a mother, I was very afraid and panicked When my son presenting with dystonia and frequent falls. After many times seeking medical attention, I learned this was a genetic disease and no effective treatment was available, I felt very hopeless, but never given up, I have been paying attention to this information, and hoping that there would be a better treatment that can help my child.

## Supplementary Information

**Additional file 1: Figure 1.** Brain MRI was normal at 7 years old. MRI. (A, sagittal T1; B, axial FLAIR). **Figure 2.** EEG was normal at 7 years old.

**Additional file 2: Table.** Neurodevelopmental phenotype with dystonia of patients with IRF2BPL variants.

## Data Availability

The datasets used during the current study are available from the corresponding author on reasonable request.

## Abbreviations

IRF2BPL: Interferon regulatory factor 2 binding protein-like
MRI: Magnetic resonance imaging
EEG: Electroencephalogram
CPAP: Continuous positive airway pressure
