# Supplementary material for: IRF2BPL gene variants with dystonia: one new Chinese case report
Source: BMC Neurol. 2023 Jan 21;23:32. doi: 10.1186/s12883-023-03077-x (PMC9862514; doi:10.1186/s12883-023-03077-x)
Supplement: Supplementary file 1 — Additional file 1: Figure 1. Brain MRI was normal at 7 years old. MRI. (A, sagittal T1; B, axial FLAIR). Figure 2. EEG was normal at 7 years old. [file 12883_2023_3077_MOESM1_ESM.pdf]

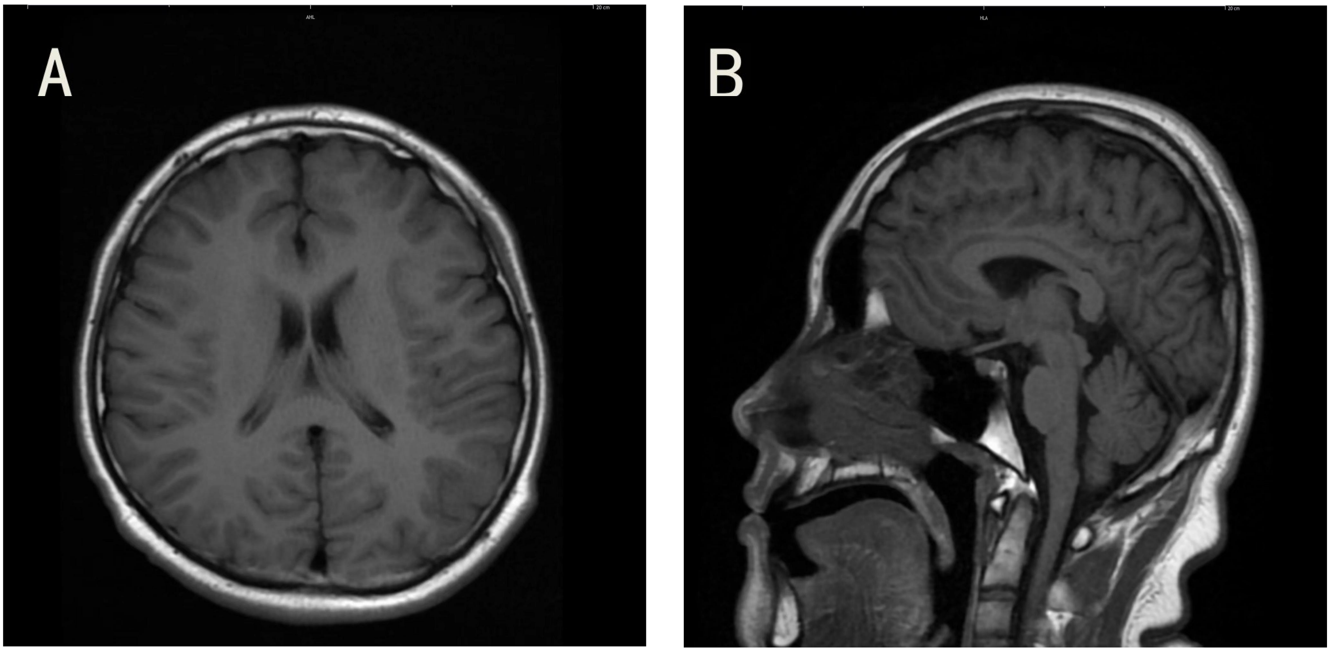

Figure1: Brain MRI was normal at 7 years old. MRI. (A, sagittal T1; B, axial FLAIR)

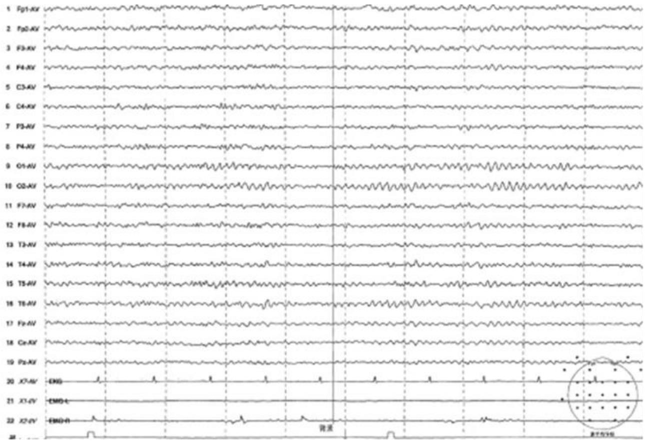

Figure2: EEG was normal at 7 years old.
