# Supplementary material for: IRF2BPL gene variants with dystonia: one new Chinese case report
Source: BMC Neurol. 2023 Jan 21;23:32. doi: 10.1186/s12883-023-03077-x (PMC9862514; doi:10.1186/s12883-023-03077-x)
Supplement: Supplementary file 2 — Additional file 2: Table. Neurodevelopmental phenotype with dystonia of patients with IRF2BPL variants. [file 12883_2023_3077_MOESM2_ESM.pdf]

Table Neurodevelopmental phenotype with dystonia of patients with IRF2BPL variants

Patient	age	IRF2BPL variant	Type of mutation	Seizure onset	Physical examination	Motor stats	Language status	Brain MRI*	Others
1	36 years	c.373C>T (p. Gln125*)	Nonsense	10 years	Cerebellar syndrome, chorea, dystonia, severe hyperreflexia	Sits up without support, ambulates with assistance	Not reported	Generalized brain atrophy with possible increased iron deposits in deep gray matter	Slow ocular saccades
2	Deceased at 12 years	c.519C>G (p.Tyr173*)	Nonsense	8.5 years	Dystonia, ataxia, choreoathetosis, spasticity, severe hyperreflexia	Nonambulatory by 4.5 years	Spoke few words; lost language by 7 years	Diffuse atrophy	Vertical oculomotor paralysis and horizontal nystagmus
3	23 years	c.376C>T (p.Gln126*)	Nonsense	7 months	Myoclonus, ataxia, tremor, generalized dystonia, cerebellar dysarthria	Ambulatory	Speaks in short sentences. IQ 40	Normal	Slow dysmetric eye saccades
4	7 years	c.584G>T (p.Gly195Val) and c.514G>T(p.Gly172*)	Nonsense	6 years	Ataxia, dystonia, choreoathetosis, spasticity, cerebellar signs, bilateral facial palsies	Clumsiness at 2.5 years; required support at 4 years; non-ambulatory at 5.5 years	Spoke at 15 months; lost language at 6.5 years	Not reported	horizontal gaze palsy with limited vertical gaze
5	Deceased at 15 years	c.562C>T (p. Arg188*)	Nonsense	None	Dystonia, lower extremity spasticity, dysarthria	Ataxia at 5–6 years. non-ambulatory by 10 years, unable to use hands by 11 years	Loss of fluency (though intact cognition) at 9 years, complete loss of language at 11 years	(8 years) mild cerebellar atrophy, small cerebellum and “bulky” corpus callosum	Continuous CPAP# requirement, Loss of bowel/bladder control by 8 years, sialorrhea, progressive Feeding intolerance
6	16 years	c.379C>T (p.Gln127*)	Nonsense	6 years	Dystonia	Can walk for short periods	Lost language by 12 years	(6 and 13 years) Normal; (15 years) thinning of corpus callosum	Attention deficit hyperactivity disorder
7	43 years	c.376C>T (p.Gln126*)	Nonsense	10 years	Hypotonia, ataxia, spastic rigidity, athetosis, dystonia, dyskinesia, hyperreflexia	Walked at 18.5 months; ataxia at 15 years; non-ambulatory 28 years; bed-ridden at 35 years due to severe dystonia	Functional status not commented on; however, lost language by late teens/20s	(34 years) global atrophy thinning of corpus callosum	-
8	Deceased at 54 years	c.581_599del, p. Gly194Alafs*12	Frameshift	None	Muscular tension, ataxia, dystonia	(19 years) walking on toes; (42years) wheelchair-bound	unable to speak a few years into the disease	Striatal atrophy	(6years) Bilateral keratoconus

9	50years	c.584delG; p. (Gly195Alafs*17)	Frameshi ft		dystonia，extrapyramidal and pyramidal signs	gait ataxia	severe dysarthria	Not reported	cognitive and psychiatric symp- toms
10	24 years	c.364C> T, p.Gln122Ter	Nonsense	22years	multifocal myoclonic jerks, intention tremor in the four limbs and gait ataxia	frequent falls	Not reported	bilateral gliotic lesions.	impairment in long-term verbal memory
11 （ours ）	10 years	C.562C>T(p.Arg188*)	Nonsense		Dystonia, hyperreflexia, dysarthria,	Decline in gait by 8 years, now nonambulatory	Lost language by 9.5 years	(8 years) Normal	Sialorrhea
